# Association of Meal Timing with Sleep Quality and Anxiety According to Chronotype: A Study of University Students

**DOI:** 10.3390/clockssleep6010011

**Published:** 2024-03-11

**Authors:** Cristina Souza da Silva Luz, Ana Elizabeth Teixeira Pimentel da Fonseca, Jefferson Souza Santos, John Fontenele Araujo, Leandro Lourenção Duarte, Claudia Roberta de Castro Moreno

**Affiliations:** 1 Department of Health, Life Cycles, and Society, School of Public Health, University of São Paulo, São Paulo 01246-904, Brazil; cristinaluz@usp.br (C.S.d.S.L.); anaelizabethts@hotmail.com (A.E.T.P.d.F.); jeffersonsouza@usp.br (J.S.S.); 2 Department of Theory and Foundations of Education, Education Sector, Federal University of Paraná, Curitiba 80230-130, Brazil; 3 Department of Physiology and Behavior, Federal University of Rio Grande do Norte, Natal 59078-900, Brazil; johnfontenelearaujo@gmail.com; 4 Department for Health Sciences, Federal University of Recôncavo da Bahia, Cruz das Almas 44380-000, Brazil; duartleandro@gmail.com

**Keywords:** chrononutrition, sleep quality, meal time, chronotype, students

## Abstract

There are several determinants of mental health symptoms, ranging from individual characteristics to social factors. Consistent with patterns in the general population, students with evening characteristics tend to exhibit more anxiety symptoms and poorer sleep quality compared to morning students. Meal timing also appears to affect sleep and may be associated with mental health symptoms. In this context, the aim of the present study was to investigate the association of the timing of the main and last meals of the day with sleep quality and anxiety levels, according to the chronotype of university students. This study was conducted in colleges in São Paulo, Brazil, and involved application of a questionnaire to 162 university students. The questionnaire collected sociodemographic information meal and study times, and included scales assessing eveningness and morningness, sleep quality, and anxiety. Students demonstrating a phase delay in both chronotype and dinner timing exhibited higher levels of anxiety compared to morning-type students. Although no associations were observed between meal timing and sleep quality, sleeping later was associated with poorer sleep quality. The study suggests that evening students and those who eat late at night are more prone to presenting mental health symptoms. More studies are needed to further investigate this association.

## 1. Introduction

Circadian rhythms are involved in several aspects of an individual’s life. These rhythms represent the frequency of physiological, behavioral, and biochemical events that occur in the human body over a 24 h period, in synchrony with the sleep–wake cycle. This cycle, in turn, is synchronized with the alternation between light and dark during 24 h and is internally synchronized, as occurs with melatonin secretion. However, it is important to note that the sleep–wake cycle can undergo temporal shifts according to age. Additionally, there is interindividual variation in the acrophase of several biological rhythms and preferences for the timing of sleep onset and wake-up, referred to as an individual’s chronotype. Thus, individuals with a morning chronotype (morningness) prefer to both wake up and sleep early, and generally exhibit better physical and mental performance in the morning. By contrast, evening-type individuals (eveningness) prefer to both wake up and sleep later, typically reaching their peak mental and physical performance in the late afternoon/early evening [1,2,3].

Sleep duration, chronotype, and sleep quality are significant determinants of adolescents’ mental health [4]. Most classes at universities and schools take place in the morning, yet most adolescents and young adults have a biologically based sleep-phase delay. The evening chronotype at this stage of life can be a problem when these individuals are required to engage in early school/college schedules [5]. Furthermore, evening-type individuals tend to nap more during the day and may be more susceptible to emotional disturbances compared to morning-type individuals [6,7].

Tan et al. [8] suggested that evening-type students tend to be unhappier than morning types. In another study [9], high levels of anxiety and poor sleep quality were observed among evening-type students. Increased anxiety among students can be related to several factors, including chronotype. Age is associated with changes in chronotype, where the chronotype is later for younger individuals, becoming an increasingly morning chronotype with advancing age; hence, it is expected to find more evening chronotype adolescents than morning chronotype ones [10]. Adolescents with an evening chronotype tend to exhibit more anxiety than their morning chronotype counterparts [11]. Generally, there is a higher prevalence of anxiety in younger age groups and females compared to older individuals and males [12]. Evening-type young adults are more likely to have anxious temperaments, while morning-type young adults are more prone to have a hyperthymic temperament [11,13]. A study of medical students conducted in Asia revealed that 54.5% exhibited anxiety [14]. These anxiety-related findings are not limited to medical students, where undergraduates in general also exhibit high anxiety levels [9].

Meal timing can influence circadian rhythms, with emerging evidence suggesting that human molecular clocks may be regulated by feeding schedules, thereby synchronizing peripheral clocks [15]. These peripheral clocks coordinate environmental, metabolic, and behavioral cues, highlighting the significant impact of meal timing on metabolism and health [16]. Specific nutrients and meal times can also modulate the circadian timing system [15]. For instance, research by Wright et al. [17] demonstrated that evening caffeine consumption delays the human circadian melatonin rhythm and lengthens the circadian period of molecular oscillations [in vitro/in vivo]. In a recent population-based study involving over 20,000 participants, we observed a link between later meal times and increased obesity risk [18].

These studies are particularly interesting for night workers, as they remain awake throughout the night. However, the question of whether night workers should eat during their shifts remains contentious [19]. In a study conducted by Crispim’s group [20], various scenarios involving fasting and standardized meals for shift workers revealed that fasting during the night shift led to heightened energy and macronutrient intake in the early morning and throughout the day, alongside lower insulin levels and HOMA-IR in the morning. This suggests that consuming a controlled-calorie meal at night may be a more effective strategy for these workers, aligning with Gupta and colleagues’ proposal of a small snack comprising 10% of the daily energy requirement as a practical option. They argue that such a snack could provide essential energy for work and enhance the pleasurable aspects of eating, crucial for regulating food intake [21]. Moreover, the consumption of healthy foods has been associated with better sleep quality, while the intake of processed and free sugar-rich foods has been linked to poorer sleep [22]. Also, effects on the quality and quantity of sleep can be observed with the consumption of diets rich in foods that may influence serotonin and melatonin activity [23]. Furthermore, some studies have linked diet to anxiety levels, such as the association of anxiety with vitamin deficiencies, specifically vitamin B6 [24]. There is also evidence that dietary-related reductions in anxiety occur, for example, after interventions with probiotics. These findings may be related to the beneficial effect of probiotics on the gut–brain system [25].

However, in a review of the relevant literature, no studies investigating the relationship between meal timing and anxiety were found. Although some evidence is available on the effect of nutrients or meal timing on circadian rhythms, chrononutrition is a relatively unexplored area [26].

In this context, the aim of the present study was to analyze the association of meal timing and sleep quality with anxiety levels according to the chronotype of university students. This study sought to test the hypothesis that morning chronotype students, who have earlier meal times, exhibit better sleep quality and lower levels of anxiety than evening chronotype students.

## 2. Results

A total of 162 students participated in the study. The mean age of the students was 22.2 ± 5.2 years. The youngest student was 17 years old, while the oldest was 55 years old. The majority of students were female (90%).

Students from several courses participated in the study, with Nutrition (41%), Biomedicine (15%), and Nursing (14%) being the predominant courses. Only 11.7% of students were attending in-person classes at the time of data collection due to the COVID-19 pandemic. The second year of college was identified as the median for the study sample. Of the students assessed, 93% were single.

Most of the students’ families owned their homes (87.7%), and the breadwinner of the household had completed higher education (67.3%). The predominant income level was three or more minimum wages. Of the sample of 162 students, 66.7% reported engaging in physical activities, where the main frequencies reported were more than three times a week (33.3%) and three times a week (16%), with 9.3% performing physical activities only once a week and 15.4% twice a week. Mean weight reported by students was 66.7 ± 16.9 kg, and mean Body Mass Index (BMI) was 22.4 ± 3.95 kg/m^2^.

The students’ routines, based on their course study periods, are presented in Table 1, showing mean times of main and last meals of the day, along with the mean wake-up time and mean start time of the first class of the day (Table 1).

With regard to students’ sleep, 67.3% reported no health problems or sleep disorders. However, 32.7% reported one or more symptoms, with the most common being sleep problems (such as insomnia), excessive sleep, and endocrine problems. Also, 56.8% of the students were not in use of any medications, whereas the remainder used one or more medications, primarily contraceptives and hormone replacements (thyroxine).

**Figure 1:**
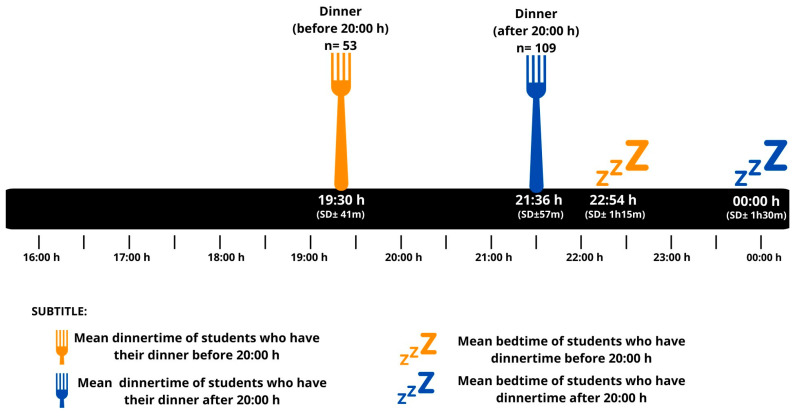
Mean dinnertime (last meal) and bedtime, stratified by students who dine before and after 20:00 h.

Concerning eating behavior, the majority of students had breakfast every day (68.5%), 23.5% only a few days a week, 2.5% only on weekends, and 5.6% never had breakfast. Students who considered breakfast their main meal woke up earlier than students who considered lunch their main meal. However, most students (73%) considered lunch their main meal. Those who had dinner before 20:00 h went to bed earlier than those who had dinner after 20:00 h. The meal times of students who dined later were associated with later bedtime hours (r = 0.51; *p* ≤ 0.001) (Figure 1). Going to bed late was associated with poor sleep quality (r = 0.22; *p* = 0.007). Additionally, earlier dinner times were associated with the morning type, while later dinner times were associated with the evening type (r = −0.43; *p* ≤ 0.001). Having lunch early was associated with the morning type (r = 0.32; *p* ≤ 0.001).

The majority of students reported poor sleep quality (71%), followed by good sleep quality (17%). Overall, 12% of participants presented signs suggestive of sleep disorder, all of whom were female. Analysis by gender revealed that 23% of male students reported good sleep quality and 77% poor sleep quality, whereas 17% of female students reported good sleep quality, 69% poor sleep quality, and 14% sleep disorders.

Based on tertiles, 36% of students were classified as evening types, 33% as intermediate, and 31% as morning types. Additionally, 9% reported going to bed at 20:00–22:00 h, 59% at 22:00–12:00 h, 24% at 12:00–2:00 h, and 8% at 2:00–4:00 h. Regarding wake-up times, 12% reported waking up at 3:00–6:00 h, 61% at 6:00–8:00 h, 19% at 8:00–10:00 h, and 8% at 10:00–15:00 h.

Concerning anxiety levels, trait anxiety, related to an individual’s personality, was higher than state anxiety, defined as a transient reaction related to a specific adverse event an individual experiences at a given time, with 17% of the students exhibiting high trait anxiety and 13% high state anxiety, while 19% had low trait anxiety and 32% low state anxiety. Furthermore, 64% had moderate trait anxiety and 55% moderate state anxiety.

For gender, moderate and high trait anxiety rates (84.2%) were found to be the greatest among female students (Table 2), this difference was not found for state anxiety (Table 3). In terms of age group, the high trait anxiety rate (25%) was the greatest in students aged 17–20 years. Students who were not physically active had a high level of both trait and state anxiety (trait: 25.9%; state: 24.1%). A moderate (53.8%) or high (46.2%) trait anxiety rate was observed in students who reported going to bed later (2:00–4:00 h). Moderate and high trait anxiety was more frequently reported by students with poor sleep quality (86%) than those with good sleep quality (64%).

**Figure 2:**
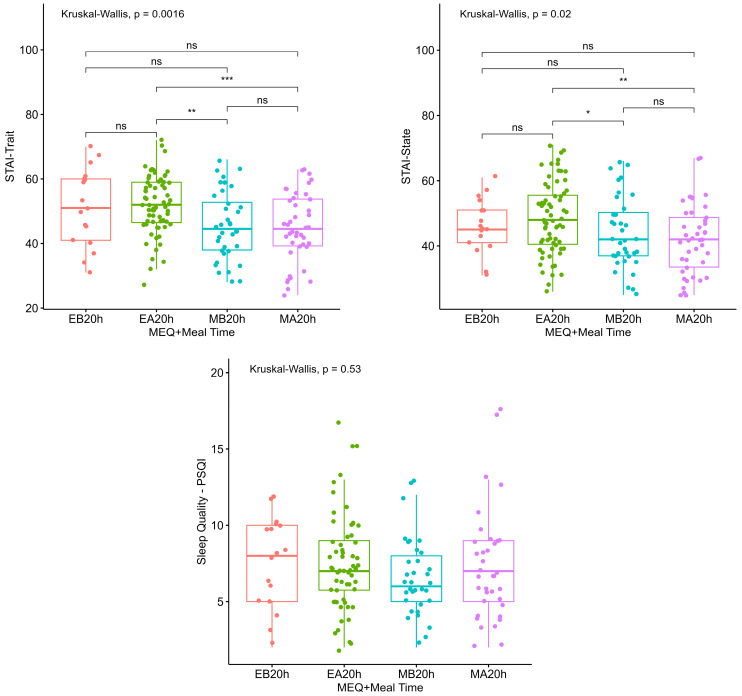
Anxiety and sleep quality according to chronotype and time of last meal. MEQ: Horne-Ostberg Morningness-Eveningness Questionnaire (chronotype). STAI: State-Trait Anxiety Inventory. PSQI: Pittsburgh Sleep Quality Index. EB20h: Evening types who eat before 20:00 h; EA20h: evening types who eat after 20:00 h; MB20h: morning types who eat before 20:00 h; MA20h: morning types who eat after 20:00 h. Significance level: *p* > 0.05; *: *p* ≤ 0.05; **: *p* < 0.01; ***: *p* < 0.001. ns: non-significant.

**Figure 3:**
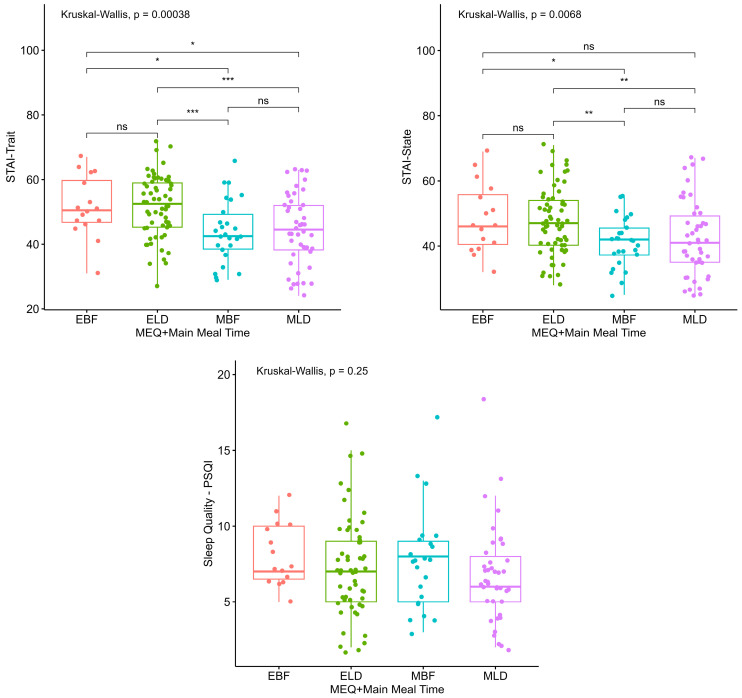
Anxiety and sleep quality according to chronotype and main meal time. MEQ: Horne-Ostberg Morningness-Eveningness Questionnaire (Chronotype). STAI: State-Trait Anxiety Inventory. PSQI: Pittsburgh Sleep Quality Index. EBF: Evening types who reported breakfast as main meal; ELD: evening types who reported lunch/dinner as main meal; MBF: morning types who reported breakfast as main meal; MLD: morning types who reported lunch/dinner as main meal. Significance level: *p* > 0.05; *: *p* ≤ 0.05; **: *p* < 0.01; ***: *p* < 0.001. ns: non-significant.

The results of grouped analyses of the independent variables (chronotype + meal times) for anxiety and sleep quality are depicted in Figure 2 and Figure 3 respectively. Evening types who reported having their last meal after 20:00 h had higher trait and state anxiety scores compared to morning types, regardless of the meal time in this group. Furthermore, the sleep quality of morning and evening types did not differ for reported meal times (Figure 2).

The analysis of trait anxiety by main meal groupings (lunch/dinner versus breakfast) showed that the evening group differed from the morning group for both meal time situations (Figure 3). A similar pattern was found for state anxiety, although no difference was found between evening individuals whose main meal was breakfast and morning individuals whose main meal was lunch/dinner. However, the evening group participants did not differ from each other in terms of trait or state anxiety, regardless of the reported time for the main meal. There was no statistical difference between the groups for sleep quality (Figure 3).

## 3. Discussion

The results of the present study partially confirmed the study hypothesis, in as far as students identified as a morning type showed lower levels of anxiety (trait and state), regardless of the time of their last meal, compared to evening types who had their last meal after 20:00 h. A similar result was observed when analyzing anxiety in relation to the main meal, as morning types showed lower levels of anxiety regardless of whether breakfast or lunch/dinner was their main meal of the day. However, although these individuals had their meals earlier than evening types as predicted, no associations were found between sleep quality and meal timing, regardless of chronotype. Budkevich et al. [27] found similar results in students who consumed food after 22:00 h and showed higher anxiety and sleep disturbances than students who ate earlier. Irregular meal timings, such as insufficient latency between the last meal and the onset of sleep and an increase in snack consumption, were also identified as markers associated with subjective mental health problems [28]. Late eating may also be associated with cardiometabolic risk factors [29], but there are few studies demonstrating the relationship among meal timing, anxiety, and sleep quality in students.

Higher levels of trait anxiety were reported by students who slept later, especially after 2:00 h, and by students who had poor sleep quality. Students who reported not engaging in regular physical activity had higher levels of moderate/high trait anxiety. These results are supported by a previous study involving students that showed a relationship between sleep quality and anxiety, as well as a high prevalence of anxiety in physically inactive students [30]. Poor sleep quality was also associated with low academic performance [31].

Female students reported higher levels of trait anxiety than males and moderate/high trait anxiety was reported predominantly by students aged 17–20 years. Other studies have also shown that women experience more anxiety than men [32,33]. Regarding age, a study of medical students aged 18–22 year conducted in Egypt reported results that contradict the present findings, showing that higher anxiety scores were significantly associated with greater age in students. This increase in anxiety with advancing age could be explained by the fact that the study population comprised medical undergraduates, a course with an increasing study load as the years progress. The present study population consisted of students with a mean age of 22.2 ± 5.2 (range 17–55) years, differing from the age profile of the cited study, and did not comprise exclusively of medical students. However, the present results corroborate the Egyptian study’s findings, which identified female gender as an important factor associated with anxiety [34].

In the present study, female students had a slightly higher prevalence of sleep problems (23%) than males (17%). These findings were similar to the results of Lund et al. [35] who, in a study of university students aged 17–24 years, found that women were more likely to report stress-related sleep problems than men. Sleep quality was also associated with low academic performance at the end of the academic year in medical students [36]. A study by Silva et al. [9] of Brazilian students showed a prevalence of 72% poor sleep quality and 5.4% possible sleep disorders, while in the present study, the prevalence of sleep disorders was 12% and the prevalence of poor sleep quality was 71%.

In this study, no association was evident between sleep quality and chronotype. However, sleep onset time was associated with sleep quality, where students who went to bed later had worse sleep quality. Nevertheless, other studies have shown a relationship between evening chronotype and poorer sleep quality in students [9,37]. The lack of association between sleep quality and chronotype in the present investigation might be explained by the delivery of pre-recorded remote classes during the pandemic. In addition, students could access classes at any time in an asynchronous fashion, which was favorable for evening types. Thus, these students could be better synchronized with their chronotype, where previous studies have attributed the poor sleep quality associated with evening types to long sleep latency, short total sleep time, and late sleep onset. These factors can be modified during remote classes since students do not need to wake up early to commute to campuses. Evening-type students who study in the morning normally have poor sleep quality due to social jet lag, which affects academic performance and quality of life [9,36].

Most of the students in the study had breakfast every day (68.5%), a habit associated with several physiological benefits, such as a reduced risk of coronary heart disease [38]. Studies have shown that people who skip breakfast are more overweight or obese [39], among other negative factors.

Anxiety levels of the students were predominantly moderate, with 64% and 55% reporting moderate/high trait and state anxiety, respectively, whereas 17% and 13% reported high trait and state anxiety, respectively. It is important to take into account that data collection for the present study was carried out between August and September 2021, and 88.3% of the students were engaged in remote learning due to the COVID-19 pandemic, declared in March 2020. A study conducted in the United States of 556 psychology students sought to test the hypothesis that state anxiety levels increased during the pandemic, while trait anxiety levels remained stable [40]. However, the authors found that trait anxiety levels increased significantly during the pandemic. In addition to the fear of the pandemic, various reasons may have contributed to the development of anxiety in this population, such as concerns about their future careers, among others. Akin to the US study, students in the present sample also exhibited higher trait anxiety levels than state anxiety.

The present study has a few limitations, such as the fact that data collection was performed through self-reporting, rendering the data more subjective. Additionally, the sample was convenience-based.

The data collection period, although not at the height of the pandemic, may have influenced the results. However, this scenario allowed analysis of meal timings for students who were “freer” due to remote classes. If the study had been conducted at another time, the meal timing of morning and evening types would likely have been more similar due to social responsibilities and commitments. Another limitation of the study was the failure to analyze the quantity and quality of meals. Future studies assessing diet composition in other populations during normal routines outside the pandemic period, for example, can add further to the literature.

In summary, despite current knowledge on the involvement of the circadian timing system in food metabolism, especially peripheral oscillators (such as the liver and pancreas), little is known about the effects of meal timings on the mental health and sleep quality of students. Elucidating the role of factors such as meal timings, quantity and food composition can promote benefits for human health [41].

## 4. Materials and Methods

### 4.1. Ethics and Data Collection

The study was submitted to and approved by the Research Ethics Committee (CEP) of the School of Public Health of the University of São Paulo (permit number: 4.821.293). All participants remotely signed the Informed Consent Form.

A quantitative cross-sectional study was conducted, with data collected from August to September 2021. Due to the COVID-19 pandemic, data collection was conducted remotely using Google Forms. The study was publicized on health-related courses with the assistance of student representatives and course coordinators, as well as by email. The form remained open and available for five weeks.

### 4.2. Data Collection Instruments

The questionnaire collected sociodemographic data, activity/class schedules, health status, use of medications for sleep induction/deprivation, and included the following scales: Morningness-Eveningness Questionnaire (MEQ), Pittsburgh Sleep Quality Index (PSQI), and State-Trait Anxiety Inventory (STAI). Additionally, participants answered questions about the timing of their main meal and their last meal before bedtime.

The Portuguese version of the Morningness-Eveningness Questionnaire (MEQ), developed by Horne and Östberg [42] and validated by Ceolim and Menna-Barreto [43], was used to identify the students’ chronotypes. The MEQ consists of 19 multiple-choice questions about sleep habits and time preferences for specific tasks during the day. Each option is assigned a score, where a lower score indicates evening types and a higher score morning types [42]. We employed terciles to better characterize our sample regarding both trait and anxiety states. Since the sample is composed of students, the majority lean towards extreme or moderate eveningness due to their study/work routine. If we take into account the traditional classification of the questionnaire, this would hinder the analysis of proportions regarding the outcome, as we would have categories with many cases and others with few cases. This compromises the calculation of the chi-square test. In addition, we divided the data into two categories according to the median for better visualization of the data in box-plot graphs.

The Pittsburgh Sleep Quality Index (PSQI) was used to assess sleep quality and possible sleep disturbances in the month leading up to application. The PSQI questionnaire has been validated for the Brazilian population [44]. The results from this questionnaire are grouped under seven components: subjective sleep quality, sleep latency, sleep duration, habitual sleep efficiency, sleep disturbances, use of sleeping medications, and daytime dysfunction. The total score on the index ranges from 0 to 21 points, classified as follows: 0–4 points indicate good sleep quality, 5–10 points indicate poor sleep quality, and a score above 10 points indicates sleep disorders. During application of the PSQI, 15 students were excluded from the analysis due to incorrect responses to some questions in this questionnaire, giving a study sample of 147 students.

Subjective components related to anxiety were measured using the State-Trait Anxiety Inventory (STAI) [45]. This instrument assesses anxiety using two scales: trait anxiety (STAI-T), related to an individual’s personality data, representing a more stable aspect related to an individual’s propensity to experience varying levels of anxiety throughout life, while state anxiety (STAI-S) reflects a transient reaction related to a specific adversity an individual experiences at a given moment. This inventory was translated into Brazilian Portuguese by Biaggio et al. [46].

The two sub-scales (state anxiety and trait anxiety) each consist of 20 multiple-choice questions, scored separately with a minimum score of 20 and maximum of 80 for each sub-scale [47]. On this inventory, score bands of 20–39, 40–59, and 60–80 points indicate low, moderate, and high levels of anxiety, respectively [48].

Students reported the times at which they usually had their main meal. Some respondents answered by giving breakfast times, while others reported lunch times. In other words, the students defined their main meal, with breakfast time defined as meals consumed before 11:00 h and lunch after 11:00 h for students who woke up early. For students who identified 11:00 h as their main meal but woke up later, this was considered breakfast. Additionally, students were asked to report the time they typically had their last meal before bedtime.

### 4.3. Statistical Analysis

The power of the statistical analysis was calculated to verify the appropriateness of the sample size. For this purpose, the value of 0.30 was chosen as the effect size, considering alpha as 0.05, in an independent sample comparison of one-way fixed effect ANOVA. Thus, the statistical power estimated was 0.90. This approach ensured that the study was sufficiently powered to yield reliable results, providing a solid foundation for the statistical analyses conducted in the investigation. The GPower program was used to perform this calculation (V. 3.1.9.7).

For statistical analysis, Jamovi Project software (2021) version 2.2 and R software (version 4.2.3) were used. First, descriptive analyses were conducted for continuous variables (mean and standard deviation) and rates (percentages) for categorical variables. Chi-square tests were used to check for differences between the levels of trait and state anxiety. Spearman’s linear correlation coefficient was used for some descriptive analyses. A significance level of *p* < 0.05 was adopted in all tests.

The hypothesis that morning-type students have earlier meal times and better sleep quality with lower anxiety levels was tested using a non-parametric analysis of variance (Kruskal–Wallis test). The Wilcoxon multiple comparisons test was used for analyses of variance that reached statistical significance.

The independent variables (chronotype, time of last meal and main meal) were grouped into the following categories: evening students who had dinner before 20:00 h (EB20h); evening students who had dinner after 20:00 h (EA20h); evening students who reported breakfast as their main meal (EBF); and evening students who reported lunch/dinner as their main meal (ELD). The same categories were adopted for the morning group (MB20h, MA20h, MBF, and MLD). Based on the median, chronotype data were divided into two categories: evening (P0–P50) and morning (P50–P100). This criterion was essential to avoid compromising the sample size in each category, which might have affected the analysis of variance.

Studies have considered 21:00 h as late-night eating [49,50]. Additionally, a recent study with the Brazilian population found a higher risk for obesity among those who have their last meal after 21:00 h [18]. Considering that the popular time for dinner in Brazil is 19:00 h, we decided to select a time between 19:00 h and 21:00 h for stratifying the food intake schedules between groups. Thus, we chose 20:00 h as the cut-off time, opting for a more conservative approach.

## 5. Conclusions

Morning-type students, regardless of the time of their last meal at night, exhibited lower levels of trait and state anxiety compared to evening-type students who dined later (after 20:00 h). Additionally, morning types had lower anxiety levels than evening types, regardless of their meal timing preferences (breakfast or lunch/dinner). No associations were found between meal time and sleep quality, irrespective of chronotype. However, poorer sleep quality and later bedtime were associated with trait anxiety, where the later the bedtime, the poorer the sleep quality. Further studies should be conducted investigating the relationship between anxiety and sleep in healthcare students, with a focus on meal timing.

## Figures and Tables

**Table 1:** Average (24 h clock) wake-up, first class start, and main and last meal times of students, by course study period (classified according to class start time into morning, afternoon, and evening).

Course Study Period	Wake-Up Time (Work/Study Time Days)	First Class Start Time	Main Meal Time	Last Meal Time
Morning	06:42 h ± 50 min	07:42 h ± 21 min	11:42 h ± 115 min	20:36 h ± 75 min
Afternoon	07:00 h ± 66 min	13:48 h ± 30 min	13:48 h ± 284 min	20:42 h ± 48 min
Evening	07:06 h ± 72 min	18:54 h ± 17 min	11:54 h ± 177 min	20:00 h ± 98 min

**Table 2:** Sample characteristics for trait anxiety.

Variables	Trait Anxiety (%)				
	Low	Moderate	High	*X* ^2^	*p*
Gender					
Female	15.7	66.4	17.8	15.67	0.004 *
Male	57.1	28.6	14.3		
Age					
17–20 years	20.8	54.2	25.0	10.00	0.04 *
21–25 years	12.7	74.6	12.7		
>25 years	29.6	63.0	7.4		
Physical Activity					
Yes	25.9	61.1	13.0	11.62	0.003 *
No	5.6	68.5	25.9		
BMI					
<18.6	0.0	63.6	36.4	10.00	0.12
18.6–24.9	24.3	60.4	15.3		
25–29.9	14.3	71.4	14.3		
>30	0.0	72.7	27.3		
Chronotype (tertiles)					
Evening	10.3	70.7	19.0	7.25	0.12
Intermediate	18.9	60.4	20.7		
Morning	29.4	58.8	11.8		
Last Meal Time					
Before 20:00 h	28.3	52.8	18.9	5.00	0.08
After 20:00 h	14.7	68.8	16.5		
Main Meal Time					
Breakfast	19.1	69.0	11.9	1.12	0.57
Lunch/Dinner	20.4	61.1	18.5		
Bedtime					
20:00–21:59 h	40.0	53.3	6.7	13.86	0.03 *
22:00–23:59 h	18.1	66.0	15.9		
00:00–01:59 h	20.5	64.1	15.4		
02:00–04:00 h	0.0	53.8	46.2		
Wake-up Time					
03:00–05:59 h	21.1	68.4	10.5	7.12	0.31
06:00–07:59 h	21.9	62.5	15.6		
08:00–09:59 h	13.3	70.0	16.7		
10:00–15:00 h	8.3	50.0	41.7		
Sleep Quality					
Good	36.0	48.0	16.0	7.00	0.03 *
Poor	14.0	67.2	18.8		

* *p* ≤ 0.05.

**Table 3:** Sample characteristics for state anxiety.

Variables	State Anxiety (%)				
	Low	Moderate	High	*X* ^2^	*p*
Gender					
Female	30.1	56.8	13.0	6.44	0.17
Male	57.1	28.6	14.3		
Age					
17–20 years	30.5	52.8	16.7	1.62	0.80
21–25 years	33.3	57.1	9.5		
>25 years	33.3	55.6	11.1		
Physical Activity					
Yes	37.0	55.6	7.4	10.19	0.006 *
No	22.2	53.7	24.1		
BMI					
<18.6	9.1	63.6	27.3	7.49	0.28
18.6–24.9	36.1	53.1	10.8		
25–29.9	28.6	60.7	10.7		
>30	18.2	54.5	27.3		
Chronotype (tertiles)					
Evening	20.7	63.8	15.5	6.53	0.16
Intermediate	34.0	54.7	11.3		
Morning	43.1	45.1	11.8		
Last Meal Time					
Before 20:00 h	35.9	52.8	11.3	0.57	0.75
After 20:00 h	30.3	56.0	13.7		
Main Meal Time					
Breakfast	35.7	57.1	7.2	1.30	0.52
Lunch/Dinner	33.3	52.8	13.9		
Bedtime					
20:00–21:59 h	46.7	33.3	20.0	7.80	0.26
22:00–23:59 h	33.0	57.4	9.6		
00:00–01:59 h	28.2	59.0	12.8		
02:00–04:00 h	23.1	46.1	30.8		
Wake-up Time					
03:00–05:59 h	31.6	57.9	10.5	7.26	0.30
06:00–07:59 h	34.4	56.2	9.4		
08:00–09:59 h	26.7	53.3	20.0		
10:00–15:00 h	16.7	50.0	33.3		
Sleep Quality					
Good	36.0	52.0	12.0	0.53	0.77
Poor	28.7	57.4	13.9		

* *p* ≤ 0.05.

## Data Availability

Correspondence and requests for materials should be addressed to crmoreno@usp.br.
